# AB-Flu nanodrug combined with exercise intervention enhances ROS-mediated antitumor effects in melanoma

**DOI:** 10.3389/fphys.2026.1767235

**Published:** 2026-02-06

**Authors:** Yuanbing Zhou, Jinke Ma, Ziyu Zhang, Xiaodan Niu, Huiyu Yan, Jun Zhang

**Affiliations:** 1 Department of Public Foundation Teaching, Institute of Integration of Sports and Medicine, Xi’an Academy of Fine Arts, Xi’an, China; 2 School of Physical Education, Xi’an Jiaotong University, Xi’an, China

**Keywords:** exercise intervention, melanoma, ROS, synergistic therapy, tumor hypoxia

## Abstract

**Purpose:**

This study aims to investigate the synergistic effects of AB-Flu nanodrugs and exercise intervention on enhancing the antitumor effects in melanoma by improving the hypoxic tumor microenvironment (TME). The focus is on evaluating how this combination influences reactive oxygen species (ROS) levels, inhibition of melanoma *in vivo*.

**Methods:**

An intact B16F10 melanoma mouse model was established, and mice were divided into four groups: control, AB-Flu treatment, exercise intervention, and combination therapy (AB-Flu + exercise). AB-Flu nanodrug was administered intraperitoneally, while exercise was facilitated by a weighted swimming intervention. Tumor growth, tumor hypoxia, ROS levels, and apoptosis were analyzed through tumor volume measurements, histological staining, and ROS detection assays. The antitumor effects of different treatments were compared.

**Results:**

The combination therapy group showed the most significant tumor inhibition efficacy with tumor growth inhibition rates of 56%, compared to 30% for the AB-Flu monotherapy group and 41% for the exercise group. Additionally, tumor tissues from the combination group exhibited significantly lower levels of hypoxia and enhanced tumor cell apoptosis. ROS levels were substantially higher in the combination therapy group compared to other groups, indicating that the combination of AB-Flu and exercise synergistically elevated ROS production, which may contribute to increased tumor cell apoptosis. No significant toxicity was observed in major organs.

**Conclusion:**

The combination of AB-Flu nanodrugs and exercise intervention significantly enhances the antitumor effects in melanoma by improving the hypoxic TME, elevating ROS levels, and promoting apoptosis in tumor cells. This strategy may offer a potential approach for melanoma therapy.

## 1 Introduction

Melanoma is a highly aggressive form of skin cancer, and its global incidence continues to rise. According to the World Health Organization (WHO), there were approximately 331,647 new cases of melanoma and 58,645 deaths worldwide in 2022 (Bray et al., 2024; Mattiuzzi and Lippi, 2019). Despite the overall decline of cancer mortality in the United States, melanoma remains a pressing concern with 104,960 new cases and 8,430 deaths estimated in 2025, in which alarming sex and racial disparities persist (Siegel et al., 2025). Melanoma is characterized by its high invasiveness and resistance to conventional therapies, rendering it one of the most pressing public health challenges worldwide. Although traditional treatments, including surgery, radiotherapy/chemotherapy, and immunotherapy, are critical in cancer management, they often present drawbacks such as adverse drug effects, psychological stress, invasiveness, poor individual adaptability, and high treatment costs (Lorusso et al., 2017; Mariotto et al., 2011; Wang et al., 2021; Zitvogel et al., 2016).

Exercise therapy has emerged as an effective adjunctive strategy in cancer treatment, improving physical fitness, enhancing immune function, and alleviating side effects, thereby assisting patients in better coping with the challenges posed by the disease and its treatment (Friedenreich et al., 2020; Hayes et al., 2023; Stout et al., 2017). Studies have demonstrated that moderate exercise not only improves the quality of life for cancer patients, but also significantly enhances antitumor immune responses by modulating the distribution and function of immune cells within the tumor microenvironment (TME), thereby inhibiting tumor growth (Christensen et al., 2018; Gustafson et al., 2021; Koelwyn et al., 2017; Sanft et al., 2023). Furthermore, during exercise, muscle contractions and increased metabolic demands result in a significant increase in levels of reactive oxygen species (ROS) (Radak et al., 2013). In tumor cells, ROS can induce oxidative stress, disrupt intracellular redox balance, and ultimately lead to cell death (Schumacker, 2006). However, the hypoxic TME confers a high tolerance to ROS in tumor cells, thereby limiting the effectiveness of exercise alone (Chen et al., 2022; Ruan et al., 2021).

Hypoxia is a critical characteristic of TME that directly influences tumor growth, metabolism, and response to treatment. Hypoxia can significantly undermine the efficacy of chemotherapy and immunotherapy by promoting angiogenesis, metabolic reprogramming, and enhancing the invasiveness of tumor cells (Koelwyn et al., 2017). Consequently, improving tumor hypoxia has emerged as a vital strategy for enhancing response rates to antitumor therapies, with current methods primarily focusing on increasing oxygen supply and decreasing oxygen consumption by tumor cells (Ban et al., 2017; Wigerup et al., 2016). Although increasing oxygen supply is theoretically feasible, it presents specific clinical limitations, particularly the potential to stimulate tumor proliferation (Lee et al., 2020). In contrast, inhibiting oxygen consumption in tumor cells by reducing mitochondrial respiration to decrease the tumor’s oxygen demand represents a more promising strategy (Buss et al., 2020; De Visser and Joyce, 2023; Dewhirst et al., 2016).

Advances in nanotechnology enable the implementation of this strategy. Researchers have developed an albumin-bound fluvastatin (AB-Flu) nanodrug that leverages the favorable biocompatibility, prolonged circulation time, and ease of modification of albumin, significantly improving hypoxic conditions in the TME by inhibiting mitochondrial respiration in tumor cells and reducing oxygen consumption (Liu et al., 2021; Lomis et al., 2016; Ma et al., 2022; Zhao et al., 2016).

Among various exercise modalities, swimming has demonstrated significant antitumor effects. Pedro et al. reported that swimming suppressed tumor progression in mice and was accompanied by decreased macrophage infiltration and reduced neutrophil accumulation (Almeida et al., 2009). In addition, another study demonstrated that swimming inhibited tumor growth and promoted tumor cell apoptosis (Yan et al., 2023). However, the suppressive effects of swimming on the hypoxic TME and tumor growth inhibition remains unknown. To investigate the suppressive effects of enhancing the hypoxic TME and exercise intervention on tumor growth and elucidate their underlying mechanisms, we established an intact B16F10 mouse melanoma model and implemented a combined treatment using the AB-Flu nanodrug and exercise intervention. Experimental results indicate that AB-Flu enhances the hypoxic TME, reduces tumor cell resistance to ROS, and further amplifies the ROS-mediated cytotoxic effects induced by exercise intervention. This combination therapy not only significantly inhibited tumor growth, but also exhibited favorable biosafety and tolerance. This study provides some of insights into the comprehensive treatment of melanoma and gives a hint for future clinical applications.

## 2 Materials and methods

### 2.1 Synthesis of AB-Flu

AB-Flu was prepared according to the previous study (Ma et al., 2022). Briefly, human serum albumin (HSA, Sigma-Aldrich, United States) (10 mg) and tris (2-carboxyethyl) phosphine (TCEP) (10 mg) were dissolved separately in 1 mL of PBS buffer to prepare a 10 mg/mL solution, followed by sonication for 10 min. Fluvastatin (Flu, Sigma-Aldrich, United States) (1 mg) was dissolved in 20 µL of DMSO buffer. Subsequently, 500 µL of the HSA solution and 500 µL of the TCEP solution were added to 10 mL of PBS buffer, along with 20 µL of the Flu solution, and the mixture was sonicated for 30 min.

### 2.2 The study strategy, cell culture and B16F10 melanoma mouse model

**FIGURE 1:**
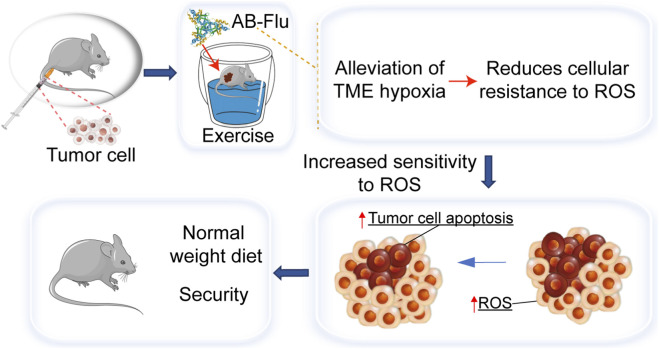
Schematic representation of tumor treatment employing the AB-Flu nanodrug in combination with exercise intervention. AB-Flu enhances the hypoxic TME, thereby decreasing tumor cell resistance to ROS. Concurrently, exercise induces an elevation in ROS levels within tumor tissues. Collectively, these interventions synergistically promote tumor cell apoptosis while ensuring favorable biosafety.

The study strategy is presented in Figure 1. The aims of this strategy are to investigate the individual efficacy of the AB-Flu and the exercise, as well as the synergistical effects of AB-Flu + exercise treatment on the hypoxic TME within B16F10 tumor tissues.

Mouse melanoma B16F10 cell line (sourced from the Chinese Academy of Sciences Cell Bank) was cultured in DMEM medium (Thermo Fisher Scientific, United States) supplemented with 10% fetal bovine serum (FBS). The culture was maintained in an incubator with 5% carbon dioxide at a temperature of 37 °C. B16F10 cells were digested and collected using 0.05% trypsin-EDTA (Thermo Fisher Scientific, United States) to obtain a sufficient number of cells.

Twenty female C57BL/6 mice (4–5-week-old) were purchased from SPF Biotechnology (Beijing, China) and housed under SPF conditions. Mice were housed using standardized breeding methods in a specific pathogen-free environment with a regular light-dark cycle. Mice were randomly divided into four groups, and subsequently, 5 × 10^5^ cells (in 100 μL PBS) were subcutaneously inoculated into the unilateral hindquarters of 4–5-week-old female C57BL/6 mice.

Following the inoculation of B16F10 cells, mice were used to evaluate the synergistic effects of AB-Flu combined with swimming intervention: the control group (Control, n = 5) receiving PBS injections; the monotherapy group (AB-Flu, n = 5) receiving intraperitoneal injections of 2 mg/kg AB-Flu; the moderate-intensity exercise intervention group (ME, n = 5) undergoing weighted swimming training; and the combination therapy group (ME+AB-Flu, n = 5) receiving both weighted swimming and 2 mg/kg AB-Flu injections. Correspondingly, mice in the control group were placed in the same swimming environment, ensuring that water levels did not interfere with their normal activities and respiration. When the average tumor volume of the mice reached approximately 50 mm^3^, PBS or AB-Flu was administered via intraperitoneal injection every 2 days. Tumor volume (V) was calculated using the formula: V = (width^2^ × length)/2. The tumor growth inhibition (TGI) rate was calculated using the formula: TGI (%) = (1-T_Treatment_/T_Control_) × 100, where T_Treatment_ represents the mean tumor volume of the treated group, and T Control represents the mean tumor volume of the control group at the same time point. After 2 weeks, mice were euthanized by manual cervical dislocation, and tumor tissues, major organs as well as the whole blood of mice were removed and collected.

All mouse experiments conducted in this study were approved by the Animal Ethics Committee of the Medical Ethics Committee of Xi’an Jiaotong University and adhered to relevant regulations. The ethical approval code is No. XJTUAE2024-2651.

### 2.3 Swimming intervention and testing

**FIGURE 2:**
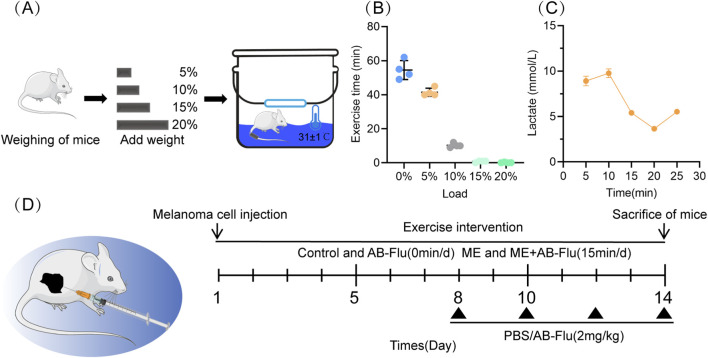
Experimental design of the weighted swimming training in mice and the combination intervention protocol. **(A)** Schematic diagram of the experimental setup for weighted swimming training in mice. **(B)** Assessment of swimming endurance in mice under various weight conditions, with a load of 5% of body weight selected for subsequent experiments. **(C)** The blood lactate concentrations in mice during 5% body weight swimming training at indicated time points. **(D)** Subcutaneous inoculation of 5 × 10^5^ B16F10 cells into the unilateral hindquarters of C57BL/6 mice, which were randomly divided into four groups: control group (Control), AB-Flu treatment group (AB-Flu), moderate-intensity exercise intervention group (ME), and the combination therapy group (ME+AB-Flu) (n = 5/group). A schematic diagram of the treatment regimen is also provided.

As the intensity swimming with 5% body weight loading is relatively low, and mainly provides energy through the aerobic metabolism system (Yan et al., 2023). In this study, the weighted swimming as the exercise intervention was performed. Swimming training was conducted in a transparent water tank measuring 30 cm in height and 25 cm in diameter, with a maintained water depth of 20 cm and a temperature of 31 °C ± 1 °C (Figure 2A) (Jones, 2007). Preliminary swimming experiments were performed with various loads. A load equivalent to 5% of the mouse’s body weight was identified as an appropriate and tolerable weight (Figure 2B), which was utilized in subsequent animal experiments.

### 2.4 Quantitative determination of plasma lactate via enzymatic-colorimetric assay

Lactate concentrations were quantified using a Lactic Acid Assay Kit (Catalog No. A019-2-1, Nanjing Jiancheng Bioengineering Institute, China) following the manufacturer’s instructions. Briefly, 0.1 mL of EDTA-anticoagulated murine whole blood was mixed with 0.6 mL of ice-cold protein precipitant (pre-chilled to 4 °C), vortexed for 30 s, and then centrifuged at 4,000 × g for 10 min at 4 °C to collect the supernatant. The supernatant was incubated with a freshly prepared enzyme working solution (lactate dehydrogenase (LDH)-NAD^+^ system) and chromogenic agent (nitroblue tetrazolium/phenazine methosulfate, NBT/PMS) at 37 °C for 10 min, under light-protected conditions. The reaction was terminated by adding 50 μL of termination solution, and absorbance was immediately measured at 530 nm using a Thermo Scientific™ Multiskan™ GO microplate reader (1 cm light path). Lactate concentrations were calculated using a six-point standard curve (0–20 mmol/L, *R* ^2^ > 0.99) generated with kit-provided lactate standards.

According to standards established by the American College of Sports Medicine (ACSM) (Garber et al., 2011), sustained blood lactate levels exceeding 4 mmol/L combined with a clearance rate of 0.8–1.2 mmol/L/min are typically indicative of high-intensity interval training (HIIT). In the present study, however, the lactate concentration decreased to 5.41 mmol/L at the 15-min mark, suggesting that the mice predominantly utilized aerobic metabolism—consistent with moderate-intensity exercise. The observed clearance rate of 0.87 mmol/L/min between 10 and 15 min approaches the range associated with HIIT but is not entirely congruent with it. Furthermore, the metabolic threshold for high-intensity exercise in rodents, characterized by peak lactate levels of 8–12 mmol/L (Bangsbo et al., 1994), was not fully reached in this experiment. These observations further justify the selection of the 15-min time point as representative of moderate-intensity exercise.

### 2.5 Hematoxylin and eosin (H&E) and immunohistochemistry (IHC) staining

Following treatment, the tumors and major organs (liver, kidney, lung, spleen, heart) were excised from the mice, fixed in formalin, and subsequently embedded in paraffin before sectioning. The sections were subjected to H&E and IHC stainings. The antibodies employed in this study were as follows: TUNEL (China Biological, China), anti-CD3 (China Biological, China), anti-CD8 (China Biological), anti-CD4 (Santa Cruz, United States), and anti-CD25 (Abcam, United States). Images were captured using a Nikon Eclipse Ni-U microscope (Nikon, Japan) (Yan et al., 2022). Five random areas from each sample were selected to assess the IHC scores. The intensity of immune staining (I) was evaluated by using the numerical score (0–3): 0-unstained, 1-weak staining, 2-moderate staining, and 3-strong staining. The immunostaining area (A) was assessed using numerical score (Bray et al., 2024; Mattiuzzi and Lippi, 2019; Siegel et al., 2025; Lorusso et al., 2017) according to the proportion of positive area: 1%–0%–10%, 2%–10%–50%, 3%–50%–90%, and 4%–90%–100%. Accordingly, the IHC score of each area was calculated as the staining intensity score (I) multiplied by the staining area score (A), i.e., IHC score = I × A (Wang J. et al., 2023).

### 2.6 ROS detection

At the conclusion of the experimental intervention, tumor tissues were collected and processed using the ROS detection kit (Nanjing Jiancheng Bioengineering Institute, China) for the mouse tumor samples. Absorbance was measured using a microplate reader (Thermo Fisher Scientific, United States) with an emission wavelength set at 525 nm and a path length of 1 cm.

### 2.7 Malondialdehyde (MDA) detection

MDA is an important biomarker for assessing the level of oxidative stress (Demir et al., 2022). At the end of the experimental intervention, tumor tissues were collected and the oxidative stress status was measured by the MDA detection kit (Beijing Solarbio Science & Technology Co., Ltd., China) from the mouse tumor samples. Absorbance was measured at 532 nm and 600 nm using a microplate reader (Thermo Fisher Scientific, United States), and MDA concentrations for each sample were calculated using the standard curve.

### 2.8 Detection of the hypoxia of tumor tissue

One hour prior to sacrifice, approximately 100 µL of Hypoxyprobe-1 solution (60 mg/kg body weight) was injected intraperitoneally into each mouse. Following sacrifice, tumor tissues were excised and subjected to routine fixation, embedding, and sectioning. Staining analysis was conducted to detect hypoxia within the tumor tissues, and representative images were captured using a Nikon Eclipse Ni-U microscope (Nikon, Japan) along with NIS Elements imaging software.

### 2.9 Blood analysis

At the end of the experimental intervention, fresh whole blood was collected from mice, processed into plasma and serum, and analyzed for complete blood counting, liver and kidney functions. Key parameters were included white blood cells (WBC), red blood cells (RBC), platelets, aspartate aminotransferase (AST), albumin (ALB), alanine aminotransferase (ALT), creatinine (Crea), and blood urea nitrogen (BUN). All blood tests were conducted in accordance with the standard clinical trial procedures established by Xi’an Jiaotong University Health Science Center.

### 2.10 Statistical analysis

All data were statistically analyzed using a two-tailed t-test, with a significance level set at p < 0.05. Results are presented as mean ± standard deviation (mean ± s.d.). Statistical analyses were conducted using GraphPad Prism software (version 9.0, GraphPad Software, United States).

## 3 Results

### 3.1 Load optimization and metabolic transition

In this study, we utilized swimming with 5% body weight loading as training model, which mainly provides energy through the aerobic metabolism system (Yan et al., 2023; Martha et al., 2024). During continuous swimming, we found three distinct phases of blood lactate dynamics (Figure 2C). In the initial Rapid accumulation phase (0–10 min), lactate level increased from 8.91 to 9.76 mmol/L (a rate: 0.17 mmol/L/min), this was followed by the compensatory clearance phase (10–15 min), during which lactate declined to 5.41 mmol/L (clearance rate: 0.87 mmol/L/min). Finally, in the Metabolic rebound phase (15–25 min), lactate level rebounded to 5.53 mmol/L. Based on these dynamics, the 15-min time point was selected as the exercise intervention period. At this time, lactate concentration fell within the moderate-intensity exercise range (4–8 mmol/L), suggesting that the mice predominantly utilized aerobic metabolism, thereby avoiding excessive fatigue and facilitating effective recovery (Ishihara and Taniguchi, 2018).

### 3.2 Exercise inhibits melanoma growth and synergizes with AB-Flu to enhance antitumor effects

To further investigate the antitumor effects of the combination of AB-Flu and exercise intervention, we established a duration of 15 min for the daily exercise intervention in an intact B16F10 syngeneic melanoma mouse model accordingly (Yan et al., 2023). These mice underwent continuous 14-day weighted swimming training, with a load equivalent to 5% of their body weight (Figure 2D). When the tumor volume reached approximately 50 mm^3^, the mice received intraperitoneal injections of either PBS or AB-Flu (2 mg/kg) every 2 days (n = 5 of each group).

**FIGURE 3:**
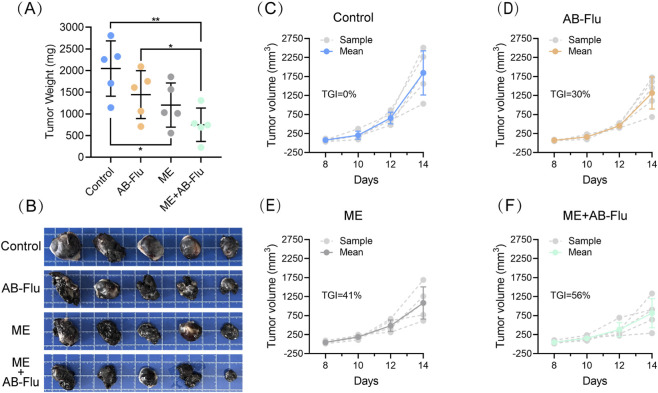
Effects of exercise intervention and AB-Flu combination treatment on the inhibition of B16F10 melanoma. **(A)** Tumor weights of each group of mice. Intergroup differences were analyzed using t-tests; *p < 0.05, **p < 0.01, ***p < 0.001. **(B)** Images of tumor tissues from each group. **(C–F)** Growth curves of tumor tissues corresponding to the control group **(C)**, AB-Flu group **(D)**, moderate-intensity exercise intervention group **(E)**, and the combination therapy group **(F)**. Data are presented as mean ± standard deviation (mean ± s.d.).

Compared to the control group, the weight (Figure 3A) and volume of tumors (Figure 3B) were significantly reduced in the AB-Flu group, ME group, and ME+AB-Flu group, demonstrating that both AB-Flu treatment and exercise intervention resulted in substantial antitumor effects. Furthermore, the tumor growth curves provide additional support for these findings (Figures 3C–F), with tumor growth inhibition rates (TGI) of 41% and 56% for the Moderate-intensity exercise intervention group (ME group) and the combination therapy group (ME+AB-Flu group), respectively. In contrast, the TGI for the AB-Flu monotherapy group was 30%. These results indicate that the antitumor effects in the combined therapy were superior to those of monotherapy and exercise intervention alone, demonstrating a synergistic enhancement.

### 3.3 AB-Flu alleviates hypoxia and enhances the antitumor effects of exercise

**FIGURE 4:**
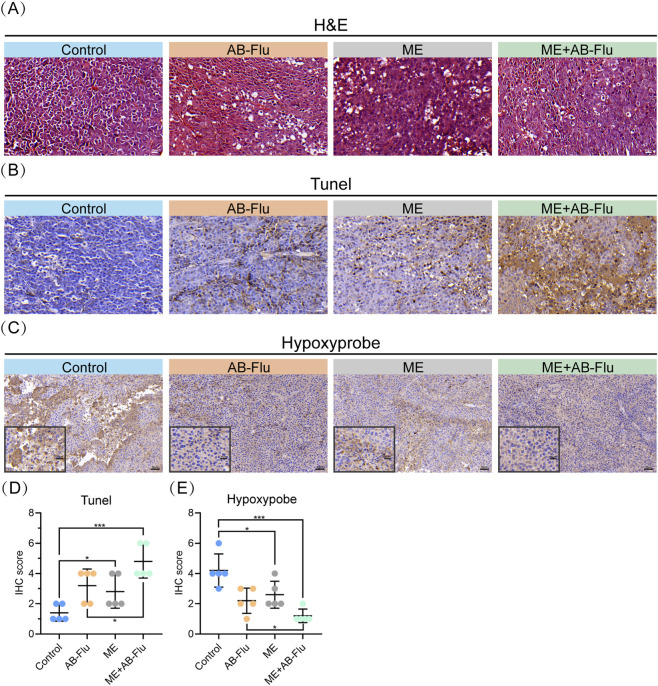
Effects of exercise intervention and AB-Flu combination treatment on hypoxia and apoptosis in melanoma tissues. **(A)** H&E-stained section images of tumor tissues from different treatment groups (scale bar: 20 µm). **(B,C)** TUNEL-stained sections (scale bar: 20 µm) **(B)** and Hypoxyprobe-stained sections **(C)** of tumor tissues from the different treatment groups (scale bar: 50 μm, enlarged drawing: 20 µm). **(D,E)** Quantitative analysis of TUNEL-stained sections **(D)** and Hypoxyprobe-stained sections **(E)** for each group of tumor tissues. Data are presented as mean ± standard deviation (mean ± s.d.). *p < 0.05, **p < 0.01, ***p < 0.001.

The combination of exercise intervention and AB-Flu therapy significantly suppressed tumor growth, highlighting the necessity of investigating internal changes within the tumor. Notably, compared to the AB-Flu monotherapy group, the ME group, and the control group, the tumor tissues in the combination therapy group (ME+AB-Flu) exhibited extensive cell necrosis and a disorganized tissue structure (Figure 4A). Although both the AB-Flu and ME groups demonstrated some degree of tumor cell destruction, the tumor tissues in the ME+AB-Flu group displayed markedly more pronounced differences. TUNEL staining and IHC scoring results indicated a significant increase in the number of positive cells in the ME+AB-Flu group, suggesting that this group exhibited the highest levels of apoptosis (Figures 4B,D). These findings indicate that exercise intervention exerts an inhibitory effect on tumor growth, while the combined use of AB-Flu further enhances this effect.

Additionally, we evaluated the hypoxic conditions in tumor tissues across the groups. The results demonstrated that the AB-Flu group significantly improved tumor hypoxia, and ME group also showed a trend toward improving tumor hypoxia. Importantly, the combination therapy group (ME+AB-Flu) exhibited the lowest levels of hypoxia in tumor tissues, indicating a synergistic effect of AB-Flu and exercise intervention in ameliorating tumor hypoxia. This finding further reinforces the connection between hypoxia alleviation and the antitumor effects of exercise (Figures 4C,E).

### 3.4 Combined exercise and AB-Flu treatment enhances antitumor effects through regulation of reactive oxygen species (ROS)

Hypoxia is a prevalent phenomenon in most solid tumors. During exercise, increased muscle contraction and energy metabolism lead to increased ROS production (He et al., 2016).

**FIGURE 5:**
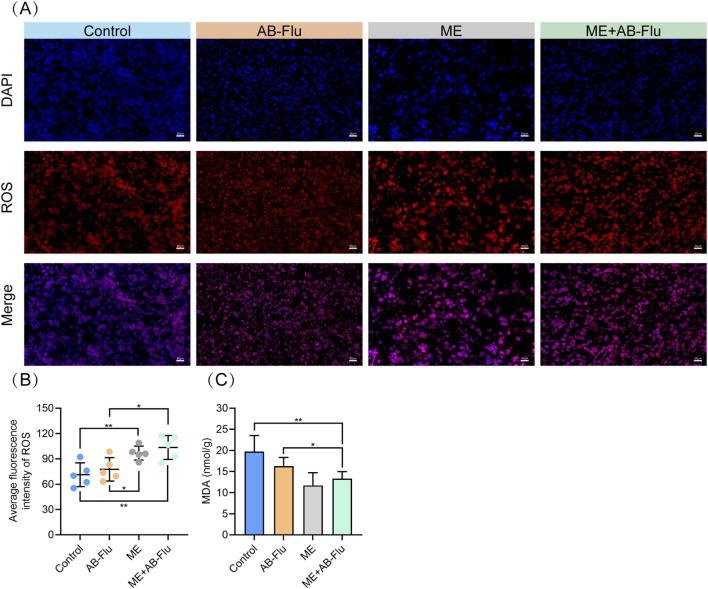
Effects of exercise intervention and AB-Flu combination treatment on reactive oxygen species (ROS) levels and malondialdehyde (MDA) content in tumor tissues. **(A,B)** Fluorescent staining of ROS in tumor tissues using a ROS probe **(A)**, with representative images selected from each group (n = 5) for quantitative analysis **(B)**. **(C)** Measurement of MDA levels in tumor tissues across groups using an MDA detection kit, reflecting oxidative stress levels. Data are presented as mean ± standard deviation (mean ± s.d.). *p < 0.05, **p < 0.01, ***p < 0.001.

To further investigate the potential mechanisms underlying the enhanced antitumor effects in the ME+AB-Flu group, we assessed ROS levels in excised tumors from the mice through immunofluorescence experiments (Figures 5A,B). As expected, ROS levels in the tumor tissues of the control group were markedly lower than those in the ME and ME+AB-Flu groups. The AB-Flu group exhibited no significant difference in ROS levels compared to the control group but differed from the ME group. These data indicate that in the ME+AB-Flu group, AB-Flu alleviated tumor hypoxia, exercise induced ROS production, which synergistically enhances the antitumor efficacy.

Furthermore, to evaluate the oxidative stress levels, we measured the MDA levels from the tumor samples of each group. The results demonstrated that MDA levels in the ME and ME+AB-Flu groups were significantly lower than those in the control and AB-Flu groups (Figure 5C). This finding indicates that exercise not only inhibits tumor growth, but also alleviates ROS-induced oxidative damage to some extent.

### 3.5 The biosafety profile of exercise combined with AB-Flu treatment

**FIGURE 6:**
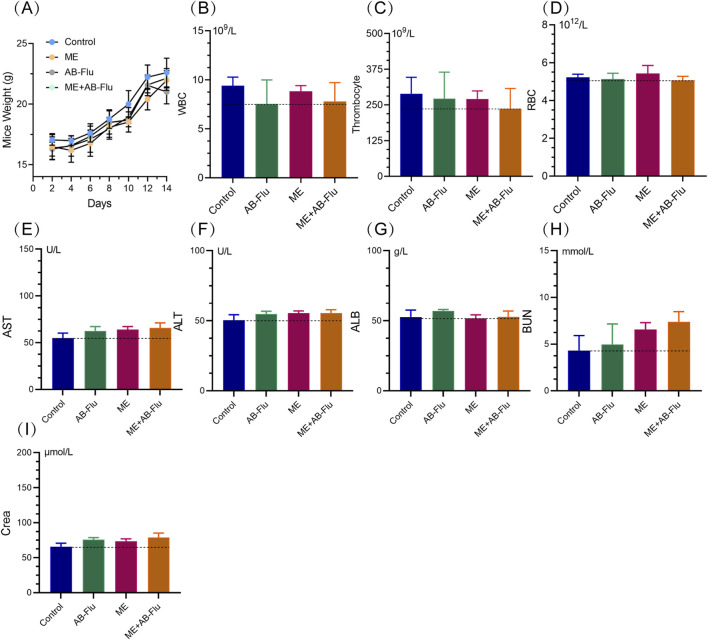
Biosafety assessment of exercise intervention and AB-Flu combination treatment. **(A)** Changes in body weight of mice across each group during treatment. **(B–D)** Routine blood parameters for each group of mice, including white blood cell count **(B)**, platelet count **(C)**, and red blood cell count **(D)**. **(E–I)** Liver and kidney function markers for each group of mice, including alanine aminotransferase **(E)**, aspartate aminotransferase **(F)**, albumin **(G)**, blood urea nitrogen **(H)**, and creatinine **(I)**. Data are presented as mean ± standard deviation (mean ± s.d.).

To investigate the biosafety of this exercise combined with AB-Flu treatment, we monitored the body weight changes, and carried out the pathological and hematological assessments of blood and major organs at the end of the study. As shown in Figure 6A, the body weights of mice in all groups exhibited no significant differences, indicating that the combination of AB-Flu and exercise intervention has minimal systemic toxicity.

Further hematological analysis (Figures 6B–I) revealed no significant changes in the routine blood parameters (such as WBC, RBC, platelets, etc.) among different groups, and liver and kidney function markers (such as ALT, AST, ALB, BUN, Crea, etc.) also showed no significant differences, suggesting that this combined treatment did not cause noticeable damage to liver and kidney functions. Although AST and Crea levels were slightly elevated in the combined therapy group, they remained within the normal range and did not cause significant toxic responses.

**FIGURE 7:**
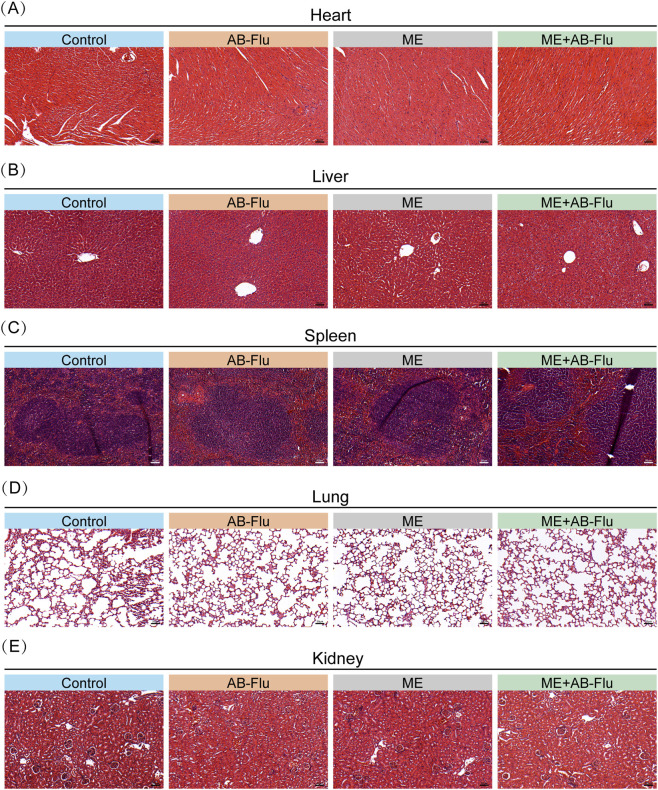
Histopathological analysis of major organs following exercise intervention and AB-Flu combination treatment. **(A–E)** H&E-stained sections of the hearts **(A)**, livers **(B)**, spleens **(C)**, lungs **(D)**, and kidneys **(E)** from each group of mice.

Additionally, the histological analysis on major organs (heart, liver, spleen, lung, and kidney) revealed no pathological changes, such as inflammation or necrosis, in the major organs of mice across all groups, and the tissue structure remained intact (Figure 7). These findings demonstrate that the combination of AB-Flu and exercise intervention exhibits good biosafety and biocompatibility *in vivo*.

## 4 Discussion

This study investigated the antitumor effects of the AB-Flu nanodrug combined with exercise intervention in an intact B16F10 melanoma mouse model. The results indicated that the combined treatment significantly suppressed tumor growth. Through a series of analyses, we confirmed that AB-Flu alleviates hypoxic conditions in tumors, and exercise induces ROS production, and the combined treatment synergistically promotes tumor cell apoptosis. Further hematological and histopathological evaluations indicated that this combined treatment did not cause significant toxic responses in the major organs of the mice, demonstrating the favorable biocompatibility.

Over the past two decades, growing research has highlighted exercise therapy as an effective adjunct for tumor. Exercise has been reported with significant improvements in patient-reported fatigue and quality of life. Preclinical studies further indicated that exercise can influence tumor incidence, progression, and metastasis. Emerging evidence showed that exercise modulates critical TME features such as hypoxia, perfusion, metabolism, and immune activity to enhance the tumor inhibition (Ashcraft et al., 2016). In particular, swimming may enhance the tumor inhibition through multiple pathways. Firstly, swimming may improve tumor perfusion, thus potentially enhancing the antitumor effects. Aerobic exercise has been reported to regulate tumor vasculature and oxygenation by promoting the formation of normal blood vessels, thereby enhancing drug perfusion within dysfunctional tumor networks (Garcia et al., 2016). McCullough et al. demonstrated that a single bout of low-to-moderate intensity exercise can increase tumor blood flow by 200% (McCullough et al., 2014). During exercise, elevated cardiac output typically directs blood flow toward metabolically active tissues. However, tumor vasculature lacks normal smooth muscle function and fails to vasoconstrict appropriately. As a result, exercise-induced high cardiac output and blood pressure lead to enhanced tumor perfusion of drugs (Thomas et al., 2020). Secondly, swimming may reshape the tumor immune microenvironment by enhancing antitumor immune activation. Physical exercise induces shear stress and adrenaline release, enhancing immune surveillance and immune modulation. Adrenaline promotes lymphocytosis, increasing circulating lymphocytes and promoting the distribution of immune cells throughout the body, which facilitating immune cell mobilization and stimulating chemokine release in TME. Gomes-Santos et al. reported that exercise increased CD8^+^ T-cell expression and the infiltration into the TME. Furthermore, exercise was shown to active CXCL9/CXCL11–CXCR3 signaling, enhancing the trafficking of CD8^+^ T cells to the tumor (Gomes-Santos et al., 2021). Esmailiyan et al. found that aerobic exercise reduced pro-tumorigenic chemokines CCL2 and CCL5 and their receptors CCR2 and CCR5, decreasing recruitment of M2 macrophages into the TME, ultimately leading to reduced tumor volume (Esmailiyan et al., 2022).

Recent developments in targeting nanodrugs provide a valuable direction for designing next-generation therapy (Yao et al., 2024). For example, Zhuang et al. reported a nanocoated *Salmonella typhimurium* platform (IF-S.T) that generates H_2_S within TME to induce self-amplified photothermal and photodynamic effects, and when combined with laser irradiation, achieves potent tumor cell killing and robust antitumor immunity leading to tumor elimination (Zhuang et al., 2024). Nanodrugs targeting metabolic pathways have also been actively explored, Qiao et al. demonstrated that a ROS-responsive, PDAC-targeted nanodrug (Z/B-PLS) enabled dual blockade of KRAS downstream PI3K/AKT/mTOR and RAF/MEK/ERK pathways to enhance photodynamic therapy (Qiao et al., 2024). In addition, Shan et al. reported that a mitochondria-targeted, ferroptosis-activated nanosystem (ICM) could remodel fatty acid metabolism and enhance ferroptosis through dual enzymatic activities and phototherapy, suppressing triple-negative breast cancer metastasis (Shan et al., 2025). Integrating such advanced nanodrugs with exercise-induced modulation may offer a highly synergistic strategy for future cancer therapy. To explore more advanced strategies, future works can study those nanodrugs targeting the TME and metabolic pathways.

Previous studies demonstrated that hypoxia is a critical feature of the TME, often leading to resistance to conventional therapies by upregulating antioxidant pathways and ROS-scavenging mechanisms (Rankin and Giaccia, 2016; Schito and Semenza, 2016; Liao et al., 2023). It is crucial to acknowledge the dual role of ROS in cancer. While elevating ROS can induce cytotoxic stress, it may also trigger adaptive antioxidant responses (e.g., Nrf2 pathway activation), thereby potentially fostering therapy resistance (Huang et al., 2021; Wang R. et al., 2023). The redox balance in cancer cells is highly sensitive, and alterations in ROS levels can produce context-dependent effects. Our results identified that AB-Flu alleviates tumor hypoxia to reduce the antioxidant buffering capacity of tumor cells, while exercise induces ROS production. These ROS levels reach the cytotoxic thresholds, thus induced apoptosis of tumor cells (Schumacker, 2006; Moloney and Cotter, 2018; Sabharwal and Schumacker, 2014). In line with these findings, our results demonstrated that ROS signals in the combined therapy group were markedly higher than those in other groups (Figures 5A,B), indicating that the synergistic effect of AB-Flu and exercise effectively disrupts the oxidative balance in tumor cells, resulting in enhanced antitumor efficacy.

Moreover, the combination treatment elevated ROS levels while preserving redox balance in normal tissues. The decreased levels of MDA indicated less lipid peroxidation and oxidative damage, which is consistent with an exercise-related enhancement of endogenous antioxidant defences (e.g., SOD, GPx), thereby mitigating lipid peroxidation reactions (Arena et al., 2019) and reducing ROS-induced oxidative stress in normal cells (Radak et al., 2013; Powers and Jackson, 2008). Although exercise induces ROS production, it simultaneously activates the body’s antioxidant defence mechanisms, which explains why the combination therapy can inhibit tumor growth while protecting normal tissues.

To further confirm the safety of this treatment strategy, we conducted a series of analysis in mice. The results indicated that the combination treatment did not cause significant side effects, either in terms of weight changes or routine blood tests and liver and kidney function markers. Even the levels of AST and creatinine were slightly elevated in the combination therapy group, they remained within the normal ranges, indicating no significant organ damage of mice. These results affirm the favorable biosafety profile of the AB-Flu and exercise combination intervention. However, several limitations should be addressed to further validate and optimize this therapeutic strategy. The use of a single mouse model (B16F10 melanoma) restricts the generalizability of the findings to other kind of dermatotumour. Additionally, the short observation period (2 weeks) limits our understanding of the long-term effects and potential chronic toxicity of the combined treatment. The small sample size in each group (n = 5 per group) may reduce the statistical power of the study, potentially masking significant differences in some outcomes. This could affect the robustness of the conclusions drawn.

Existing studies have demonstrated that exercise-induced ROS modulation exhibits antitumor potential in various cancer types beyond melanoma, including breast, colorectal, and lung cancer (Christensen et al., 2018; Koelwyn et al., 2017). For example, vigorous exercise (≥6 METs) suppresses breast cancer growth by modulating redox homeostasis, synergizing with chemotherapy or radiotherapy to enhance ROS-mediated apoptosis, and regulating ROS-related pathways via myokines (Assi et al., 2020). This finding suggests that our strategy may have applicability across multiple cancer types particularly in solid tumors with hypoxic TME and high ROS tolerance.

Regarding approved melanoma therapies, agents such as BRAF inhibitors (vemurafenib) and MEK inhibitors induce ROS accumulation by disrupting mitochondrial metabolism (Delgado-Goni et al., 2016), while immunotherapies (anti-PD-1) enhance ROS-dependent antitumor immunity (Gustafson et al., 2021). These therapies highlight that ROS modulation is a validated therapeutic target in melanoma, as it directly addresses the redox adaptability of tumor cells. Our combination strategy complements these approaches by simultaneously alleviating hypoxia (to reduce ROS tolerance) and enhancing ROS production (to exceed cytotoxic thresholds), providing a novel ROS-targeting approach with non-pharmaceutical augmentation. In addition, there are multiple existing clinically approved therapies for melanoma, such as using vemurafenib, that can also alter ROS to some extent (Teppo et al., 2017). Future studies can consider the therapeutic potential of exercise combined with such single targeted agents. Notably, vemurafenib exerts its antitumor effects by inducing ROS production to inhibit melanoma growth. Combining this agent with exercise may yield synergistic efficacy, potentially minimizing the complexity associated with multiple drug combinations, and thereby reducing adverse effects and improving patient compliance. Additionally, the present study was conducted using the B16F10 melanoma model. The efficacy of both AB-Flu + exercise combination and exercise + targeted agents (e.g., vemurafenib) in other tumors that are resistant to conventional therapies (e.g., chemotherapy, targeted therapy) remains to be validated. Given the role of ROS modulation in treatment resistance, future studies can establish drug-resistant tumor models to verify whether these exercise-based synergistic strategies can reverse resistance by disrupting the adaptive antioxidant response.

In conclusion, the combination of the AB-Flu nanodrug and exercise intervention demonstrated significant antitumor effects while exhibiting favourable biosafety. This strategy enhances tumor cell sensitivity to ROS by improving hypoxic conditions in the TME, providing a hint of seeking a potential approach for future melanoma therapies.

## Data Availability

The raw data supporting the conclusions of this article will be made available by the authors, without undue reservation.
